# How the Prevalence of Pulp Stone in a Population Predicts the Risk for Kidney Stone

**DOI:** 10.22037/iej.v13i2.18181

**Published:** 2018

**Authors:** Najmeh Movahhedian, Abdolaziz Haghnegahdar, Fatemeh Owji

**Affiliations:** a *Department of Oral and Maxillofacial Radiology, Dental School, Shiraz University of Medical Sciences, Shiraz, Iran*; b * Student Research Committee, Dental School, Shiraz University of Medical Science, Shiraz, Iran*

**Keywords:** Dental Pulp Stone, Kidney Stone, Nephrolithiasis, Pulp Calcification, Radiography

## Abstract

**Introduction:**

Conflicting researches exist on relationship between pulp stones and systemic disorders. Nephrolithiasis is a common disease with severe pain and discomfort with increasing prevalence worldwide. The purpose of this study is to evaluate the correlation between pulp and kidney stones to help find a method for early detection of kidney stones.

**Methods and Materials:**

the sample of this case-control study comprised of 154 subjects (77 patients with and 77 patients without kidney stone approved by sonographic examination). Two oral and maxillofacial radiologists evaluated their panoramic images for the presence of pulpal stones.

**Results:**

A total of 42.9% of subjects showed pulp stones. Most of the teeth with pulp stone in case and control groups were molars (86.30% and 72.97%, respectively). In the group with kidney stones, pulp stones were detected in 38 patients (49.4%), while in the control group, they were detected in 28 subjects (36.4%). Although there was not a significant relationship between the presence/absence of pulp stone and kidney stone (*P*=0.143), there was statistically significant association between number of teeth with pulp stone in a patient and the presence of kidney stone (*P*<0.013). The chance of having kidney stone is 5.78 times higher in the subjects having pulp stone in three teeth or more (≥ 3 teeth).

**Conclusion:**

Although there is not a correlation between the presence of pulp and kidney stone, the chance of having kidney stone is 5.78 times higher in the subjects with ≥ 3 teeth having pulp stone. Thus, the number of teeth with pulp stone can serve as a predictor for possibility of having kidney stone.

## Introduction

Pulp stones are definite calcified foci which are seen in coronal/radicular pulpal spaces of teeth [1]. They mostly occur in coronal pulp chamber of maxillary posterior teeth in both primary and permanent dentition [2]. Pulp stones larger than 200 µm are detectable in projectional radiographic examinations [3]. They vary in size and shape; they may be round or ovoid, single or multiple [4]. Their prevalence is highly variable in different reports, ranging from 8% to 95% [3]. Pulp stones make it difficult or impossible to approach the root canals, by narrowing their pathways which can cause failures in endodontic therapies.

Although the association of pulp stones with caries, pulpal irritations, dental restorations, periodontal diseases, epithelial rests in the pulpal tissue, aging, gender, and orthodontic movements of the teeth has been examined [2, 5-9], conflicting results on relationship between systemic disorders and pulp stones still exist in the literature.

As pulp stones are ectopic calcifications, their possible correlation with calcifications in other organs seems to be of great interest currently. In a study on the relationship between pulp and salivary gland calcification, no significant association was found [10]; while another study on evaluating the association of pulp stone with coronary artery stenosis showed positive results [11]. 

Nephrolithiasis is a common disease which its prevalence is increasing worldwide [12]. It can cause vague flank pain, hematuria, renal colic, dysuria, urinary frequency, renal colic and abdominal pain related to the region involved [13]. Today, the routine approach for detection of kidney stones is by sonographic examination [14]. Late diagnosis may lead to significant pain and dysfunction. Regarding the prevalence and undesirable consequences of these stones, a safe and available screening method for early detection would be of great help.

Dental panoramic examination is an integral part of dental check-ups. Moreover, studies showed that pulp stones can be detected well in panoramic as well as in periapical and bitewing radiographies [15-18]. The purpose of the present study was to determine the correlation between pulp stones and kidney stones in a selected population, using panoramic views. The results of this study may be helpful in early detection of kidney stones and preventing the undesirable consequences.

## Materials and Methods

Patients referred to the radiology and sonography department of Namazi Hospital in Shiraz, Iran, for evaluation of the presence of kidney stones during a one-year period, were asked to take part in the this case-control study. Among these patients, 77 subjects with documented kidney stones based on sonography and 77 subjects with sonographic approval of not having any kidney stones were selected as the study and control group, respectively. The sample size was calculated based on prevalence of pulp stone in a selected Iranian population (*P*=12%) [19] and expected value in cases (*P*=30%) at *α*=0.05 and *β*=0.20.

To be included in the study, subjects should have necessary indication for panoramic radiography, not having more than five missing/extracted teeth (including third molars) or restorative crowns and deep carious lesion/amalgam fillings that obscure pulp chamber in the radiograph. Subjects over 55 years of age were also excluded from the study, since it has been shown that the incidence of pulp stone would be higher in older patients [7, 20, 21]. The present research has been conducted in accordance with ethical principles of the World Medical Association Declaration of Helsinki (version 2008) and was approved by the Ethical Committee of Shiraz University of Medical Sciences with Approval ethics code of ir.sums.rec.1395.s799. All the subjects signed a written consent explaining the aim of the study for being included in the study.

All the panoramic images were obtained by Planmeca Intra x-ray unit (Planmeca, Helsinki, Finland) with magnification of 1.2, in Oral and Maxillofacial Radiology Department of Shiraz Dental School. Photo stimulated phosphor plates were used as image receptor, read by Regius Model 110 (Konika, Minolta, Tokyo, Japan). Two oral and maxillofacial radiologists evaluated all the images on a 17-inch computer monitor with Medecom software (Daoulas, France) in a semi-dark room for the presence of pulpal stone. The pulp stone was defined as definite radiopaque foci in the pulp chamber/canals of the teeth. Such a radiographic feature was considered as a pulp stone only if both observers were in agreement.

***Statistical analysis***

Data were described using mean±SD and frequency (percentage). The *chi*-square test, odds ratio (OR) index with 95% confidence interval (CI), and Mann-Whitney U test were used for assessing the relationship between kidney stone with pulp stone and other independent variables. A *P*-value less than 0.05 was considered statistically significant.

## Results

In the present study 154 subjects were evaluated radiographically, 77 patients with kidney stones (with the mean age of 44.32 ± 11.52 years) and 77 patients without kidney stones (with the mean age of 41.38±12.05 years). There was no significant difference between the age range of the case and control groups (*P*-value=0.124) (Table 1).

In the group with kidney stone, pulp stones were detected in 38 patients (49.4%), while in the control group, pulp stones were detected in 28 subjects (36.4%). Although there was not a significant relationship between the presence/absence of pulp stone and kidney stone (*P*=0.143), the possibility of having kidney stone was 1.7 times higher in the presence of pulp stone (OR=1.705, 0.895-3.248). At the same time, the Mann-Whitney U test showed that there was a statistically significant association between number of teeth with pulp stone and the presence of kidney stone (*P*=0.013). Moreover, the OR index demonstrated that the chance of having kidney stone is 5.78 times higher in the subjects having pulp stone in three or more teeth (95% CI: 2.013-16.592). This chance was not significantly different between those who had 1-2 teeth with pulp stone and those without pulp stone (OR=0.820, 95% CI: 0.378-1.778) (Table 2). 

Regarding the distribution of pulp stones in different tooth types, molar teeth were the most frequent tooth type with radiographically detectable pulp stone. In the group with kidney stone, among 73 teeth which had pulp stone, 63 were molar teeth (86.30%) most of which were first molars (40 first molar teeth, 54.80%). Similarly, in the control group, among 37 teeth with pulp stone, 27 (72.97%) were molar teeth (16 first molar teeth with prevalence of 43.24%) (Table 3).

Focusing on the pulp stone, the data analysis revealed that among all the patients, 66 subjects (42.9%) showed pulp stones at least in one tooth. Regarding the relationship between age and pulp stone, the result of independent *t*-test showed that the mean age of the patients with pulp stone (44.80 ± 11.56) was significantly higher than subjects without pulp stone (40.40±11.32) (*P*=0.021). The result also showed that there was no association between gender and pulp stones (*P*=0.141) (Table 4).

## Discussion

The present study evaluated the correlation between the prevalence of pulp stone in panoramic radiography with the presence of kidney stone in a selected population.

Different values are reported for prevalence of pulp stones in patients (12-72.4%) and teeth (5-19.3%) for different populations [2, 3, 6-8, 10, 19]. In the present study we found 42.9% of patients to have radiographically detectable pulp stones. However, such findings are not completely comparable because of different methods used. Most of the studies, like our study, reported the molars (specially the first molars) to be the most frequently affected teeth [2, 3, 6-8].

So, the determination of pulp stone prevalence relying on just posterior teeth, may estimate it higher than the actual value; while most of the studies used posterior bitewing or periapical radiographs for the evaluation, it seems that full mouth radiographic examination or panoramic radiography that can depict the whole dentition may be considered as more valid tools. Since studies showed that pulp stones can be detected well in panoramic radiography [15-18] and moreover, the patients dose would be lower in panoramic examination, the present study used panoramic for this purpose. 

**Table 1:** The age of subjects in case and control groups

		**Kidney stone**		
		**Present (** ***n*** **=77)**	**Absent (** ***n*** **=77)**	***P*** **-value**
**Age**		44.32±11.5	41.38±12.05	0.124
**Gender**	**Female**	40 (51.9%)	44 (57.1%)	0.517**
	**Male**	37 (48.1%)	33 (42.9%)	

* *Student t-test;*

** *chi–square test*

**Table 2:** Correlation between pulp stone/number of involved teeth with pulp stone and kidney stone

		**Kidney stone**			
		**Absent (** ***n*** **=77)**	**Present (** ***n*** **=77)**	***P*** **-value**	**OR (95%CI)**
**Pulp stone **	**Absent**	49 (63.6%)	39 (50.6%)	0.143*	1
	**Present**	28 (36.4%)	38 (49.4%)		1.71 (0.895-3.248)
**Number of teeth with pulp stone**	**0**	49 (63.64%)	39 (50.65%)	0.013^†^	1
	**1-2**	23 (29.87%)	15 (19.48%)		0.820 (0.378-1.778)
	≥3	5 (6.50%)	23 (29.87%)		5.78 (2.013-16.592)

* *: Chi-square test,*

† *: Mann-Whitney U test*

**Table 3:** Distribution of pulp stones according to different tooth types in the case and control group

**Pulp stone**			**N**	**Kidney stone**
**Anterior**	**Pre-molar**	**Molar**		
4 (10.81%)	6 (16.22%)	27 (72.97%)	37	**Absent**
1 (1.37%)	9 (12.33%)	63 (86.30%)	73	**Present**
5 (4.55%)	15 (13.64%)	90 (81.81%)	110	**Total**

**Table 4:** Distribution of pulp stone according to age and gender

		**Pulp stone**		
		**Present (** ***n*** **=66)**	**Absent (** ***n*** **=88)**	***P*** **-value**
**Age**		44.80±11.65	40.40±11.32	0.021*
**Gender**	**Female**	41 (62.1%)	43 (48.9%)	0.141**
	**Male**	25 (37.9%)	45 (51.1%)	

* *Student T- test;*

** *Chi – square test*

Sener *et al.* [22] reported that if the subjects of the study have more teeth for radiographic evaluation of the pulp stone, the result will be more reliable. In the light of this finding, the subjects were included in the present study if they had not more than five missing/extracted teeth (including third molars) or restorative crowns/deep amalgam fillings which obliterate the pulp chamber.

Since no smaller than 200 µm pulp stones can be detected in projectional radiography [3], such radiographic examinations may underestimate the prevalence of pulp stones. On the other hand, because of the higher patient dose in cone-beam computed tomography (CBCT), it is not ethical to use such a method for the evaluation of the whole dentition for the presence of pulp stone. So, projectional radiography is still the only non-invasive method for clinical evaluation for this purpose. 

Histopathologic examination of the extracted teeth has also been used for evaluation the presence of pulp stone. In a study by Aleksova *et al.* [1] extirpated pulps of teeth (without radiographic pulp stones) belonging to patients with kidney/ball calcification, were evaluated histopathologically. The result showed that all teeth had sand in pulpal area. By contrast, besides of the *in vitro* nature of histopathologic examinations, there are even some limitations for such examination. For example, this method can underestimate the prevalence of calcifications if there are not enough sections of the tooth [22].

There is a dispute in the literature regarding the correlation between pulp stones and systemic disorders. Edds *et al.* [23] indicated that a significant higher number of patients with pre-existing cardiovascular disorders had detectable pulp stones compared to a control group (74% *vs*. 39% ). They suggested that it may be helpful to use a radiographic presence of pulp stones as a screening tool for cardiovascular disorders. Similarly, Khojastepour *et al.* [24] reported a sensitivity of 68.9% for pulp stone presence in panoramic x-ray examination in this regard. Likewise was the result reported by Nayak *et al.* [25] regarding association between pulp stone and systemic diseases including cardiovascular disorders, type II diabetes mellitus and autoimmune disorders. A strong correlation between pulp narrowing and duration of kidney diseases was reported by Galili *et al*. [26].

In contrast to these findings, there are other studies which found no correlation between pulp stones and systemic disorders such as cardiovascular diseases [22]. Krell *et al*. [27] who histologically evaluated dental pulp vasculatures found no arteriosclerotic changes in atherosclerotic monkeys. In parallel, Kansu *et al*. [4] also demonstrated that there was no association between pulp and carotid artery calcification in patients with renal transplantation. However, the results of the latter study are in complete dispute with the study of Näsström *et al*. [28] that reported a positive association in this regard. 

Ertas *et al.* [3] found no association between the presence of pulp stone and kidney stone. Similar results were witnessed in the present study. But at the same time, we found that although there is no correlation between the presences of these two phenomena, strong correlation exists between the number of teeth with pulp stones and kidney stone. As if we have calcified particles in three teeth or more, it is 5.78 times more probable to detect kidney stones, too. With the mentioned limitation for projectional radiography, we may expect that with a more precise method, this correlation would be even stronger. However, in spite of this strong correlation, it would not seem appropriate to make a stringent generalizable estimation due to limited sample size. It would be helpful if a new study with a larger sample size is designed.

## Conclusion

In the present study 42.9% of subjects showed pulp stones in the panoramic view. Most of the teeth with pulp stone in the case and control groups were molars (86.30% and 72.97%, respectively). Although there is not a correlation between the presence of pulp and kidney stone, the chance of having kidney stone is 5.78 times higher in subjects with three or more teeth having pulp stone. Thus, the number of teeth with detectable pulp stone in panoramic radiography can serve as a predictor for the probability of having kidney stone. Since panoramic radiographs are routinely ordered in dental examinations, dental professionals can use them to screen patients who are at the risk of kidney stone. Beside benefit of patients, this will accentuate the brilliant roll of dentists in public health.
